# The Inventory of Personality Organisation: its psychometric properties among student and clinical populations in Japan

**DOI:** 10.1186/1744-859X-8-9

**Published:** 2009-05-06

**Authors:** Hiromi Igarashi, Hiroyoshi Kikuchi, Rikihachiro Kano, Hiroshi Mitoma, Masahiro Shono, Chieko Hasui, Toshinori Kitamura

**Affiliations:** 1 Department of Clinical Behavioural Sciences (Psychological Medicine), Kumamoto University Graduate School of Medical Sciences, 1-1-1 Honjo, Kumamoto, Kumamoto, Japan; 2 Tokyo International University, Tokyo, Japan; 3 Mitoma Clinic, Kumamoto, Kumamoto, Japan; 4 Yuge Hospital, Kumamoto, Kumamoto, Japan

## Abstract

**Background:**

The Inventory of Personality Organisation (IPO) is a self-report measure that reflects personality traits, as theorised by Kernberg.

**Methods:**

In study 1, the Japanese version of the IPO was distributed to a population of Japanese university students (N = 701). The students were randomly divided into two groups. The factor structure derived from an exploratory factor analysis among one subsample was tested using a confirmatory factor structure among another subsample. In study 2, the factor-driven subscales of the IPO were correlated with other variables that would function as external criteria to validate the scale in a combined population of the students used in study 1 and psychiatric outpatients (N = 177).

**Results:**

In study 1 the five-factor structure presented by the original authors was replicated in exploratory factor analyses in one subgroup of students. However, this was through reduction of the number of items (the number of the primary items was reduced from 57 to 24 whereas the number of the additional items was reduced from 26 to 13) due to low endorsement frequencies as well as low factor loadings on a designated factor. The new factor structure was endorsed by a confirmatory factor analysis in the other student subgroup. In study 2 the new five subscales of the Japanese IPO were likely to be correlated with younger age, more personality psychopathology (borderline and narcissistic), more dysphoric mood, less psychological well-being, more insecure adult attachment style, lower self-efficacy, and more frequent history of childhood adversity. The IPO scores were found to predict the increase in suicidal ideation in a week's time in a longitudinal follow-up.

**Conclusion:**

Although losing more than 40% of the original items, the Japanese IPO may be a reliable and valid measure of Kernberg's personality organisation for Japanese populations.

## Background

The classification and diagnosis of personality disorders have long interested clinicians and researchers. Those patients with such terminologies as pseudoneuroses and latent schizophrenia have been thought to be 'located' between neuroses and psychoses. These clinical conditions were recognised as borderline personality pathology and categorised as a personality disorder in the Diagnostic and Statistical Manual of Mental Disorders, 3rd edition (DSM-III) [1] based on their patterns of cognition, affectivity, interpersonal functioning, and impulse control. They are of particular clinical importance due to their treatment resistance. Studies of psychological therapies for borderline personality disorder have been published primarily as a compilation of cases lacking empirical data. However, a randomised control trial was recently reported [2,3].

Contrary to the descriptive approaches adopted by the DSM, Kernberg [4,5] proposed a personality structure consisting of three layers: neurotic, borderline, and psychotic. This classification was derived from psychoanalytic theory. According to Kernberg's theory, borderline personality organisation could be characterised by (1) non-specific manifestation of ego weakness, such as lack of anxiety tolerance, lack of impulse control, and lack of developed sublimatory channels; (2) a shift towards primary-process thinking; (3) specific defensive operations, such as splitting, primitive idealisation, early forms of projection and projective identification, denial, and omnipotence and devaluation; and (4) the pathology of internalised object relationships. These considerations are important because although the pathological diagnosis of personality disorders is reliably based on the behavioural descriptions detailed in the DSM, insight-oriented psychotherapies such as psychoanalysis do not target these behavioural manifestations but rather the changes in a person's in-depth personality that can only be measured using concepts matching the therapeutic theory described above.

Understanding a client's personality organisation is important when planning treatment and observing its results, but the primary means of assessing personality structure has been interviews, which are difficult to standardise [6]. Kernberg and colleagues thus developed a self-report to operationalise personality organisation: the Inventory of Personality Organisation (IPO) [7]. This instrument assesses three domains: primitive psychological defences, reality testing, and identity diffusion. To these, the authors also added two supplementary scales: aggression and moral value. The reliability and validity of the original IPO has been confirmed [8].

The present study is a preliminary report using the IPO, which we translated into Japanese, in Japanese non-clinical (undergraduate student) and clinical populations. We examined the factor structure of the inventory by both exploratory and confirmatory factor analyses. Its concurrent validity was examined by using the self-report measures of borderline and narcissistic personality disorders. We also hypothesised that psychological maladjustment (for example, negative affects including depression and anxiety, poor psychological well-being, insecure adult attachment style, low self efficacy, and history of childhood adversities) would be stronger in those with more severe borderline personality pathology. Finally, the predictive validity of the Japanese IPO was examined in terms of predicting suicidal ideation in weekly follow-up of the students.

## Study 1

### Methods

#### Participants

Students from five universities in Tokyo and Kumamoto were solicited to participate in a questionnaire survey. Usable data were available from 701 students, 172 men and 529 women. Their mean (standard deviation (SD)) age was 19.6 (2.3) years old with the range between 18 and 40. Men (mean = 20.0, SD = 2.5) were slightly but significantly (t = 2.9 *P *< 0.01) older than women (mean = 19.4, SD = 2.2). Because we asked lecturers of each university to distribute the questionnaire we were unaware of the exact number of students who were solicited. Hence we had no means to compare students who participated in the study and those who did not in terms of key variables.

#### Measurement

Personality organisation: the IPO is a self-report measure consisting of 83 items on a 5-point scale from 'never true = 1' to 'always true = 5'. This tool was developed based on the central dimension of Kernberg's [5] personality organisation model: primitive psychological defences, identity diffusion, and reality testing. These dimensions are measured by the 3 primary scales of the IPO: Primitive Defences (16 items), Identity Diffusion (21 items), and Reality Testing (20 items). Added to these are 2 additional scales, Aggression (18 items) and Moral Values (8 items with 2 Primitive Defences items and 1 Identity Diffusion item). The psychometric properties of the original IPO have been reported previously [8,9]. With the original author's permission, we translated this inventory into Japanese. In order to verify the accuracy of the Japanese translation, a translator unfamiliar with the original document back-translated the Japanese version into English.

#### Procedure

All the questionnaires were anonymously distributed and collected in a university class. This project was approved by the Ethical Committee of Kumamoto University Graduate School of Medical Sciences.

#### Statistical analysis

First, we examined the means and SDs of all the IPO items among the 701 students. Then after randomly dividing the students into two groups, we performed a series of exploratory factor analyses (EFA) separately for the primary and additional items of the IPO using data from one group of students. Because inclusion of items with a low base rate in an EFA may cause distorted structure, we excluded items from analyses if their mean was less than 1.4, which is 1/10th from the lowest score of 1 within a range of 4 (Table 1). All factors were considered dependent upon each other. The factor solution was sought after promax rotation, which is a diagonal rotation.

**Table 1:** Means and standard deviations (SDs) of the Inventory of Personality Organisation (IPO) items (N = 353)

No.	Category	Question	Mean	SD
1	PD	I am a 'hero worshiper'	1.82	1.00

2	PD	People I once thought highly of have disappointed me	2.38	1.01

3	PD	It has been a long time since anyone taught me anything I did not know	1.99	1.02

4	PD	People turn against me or betray me	1.83	1.05

5	PD	I admire people in order to feel secure	2.54	1.08

6	PD MV	I do things that at other times I think are not too wise	1.90	1.09

7	PD	I have difficulty in seeing shortcomings in those I admire	1.27	.061

8	PD	I don't get what I want	2.50	1.22

9	PD	I behave in contradictory ways	1.83	1.01

10	PD	People are basically either good or bad	1.78	1.06

11	PD	People use me	1.75	0.98

12	PD	I act in unpredictable and erratic ways	2.45	1.15

13	PD	I have favourite people whom I idealise	2.62	1.27

14	PD MV	I do things that I later find hard to believe I did	2.59	1.05

15	PD	People either overwhelm me with love or abandon me	1.62	0.96

16	PD	I feel things with either joy or despair	2.22	1.19

17	ID MV	Others see me as quite different from the way I really am	1.71	1.05

18	ID	I'm different at home than I am at work/school	2.46	1.33

19	ID	My tastes and opinions are borrowed from other people	1.86	1.03

20	ID	I behave differently in different situations	2.41	1.22

21	ID	I fluctuate between being warm and cold	2.77	1.23

22	ID	I provoke people to get my way	1.41	0.79

23	ID	I can't explain the changes in my behaviour	1.93	1.14

24	ID	I do things on impulse that are socially unacceptable	2.14	1.07

25	ID	It's hard for me to say no	2.35	1.18

26	ID	My life seems like a series of short stories	1.96	1.24

27	ID	I pick up interests and then drop them	2.17	1.16

28	ID	When others see me as having succeeded, I'm elated	2.79	1.31

29	ID	Important people suddenly change their attitudes towards me	3.36	1.31

30	ID	It is hard for me to be sure about what others think of me	3.30	1.27

31	ID	Being alone is difficult	2.10	1.14

32	ID	I see myself in different ways at different times	2.60	1.23

33	ID	In an intimate relationship, I'm afraid of losing a sense of myself	1.87	1.18

34	ID	My life goals change frequently	2.25	1.19

35	ID	My goals keep changing	2.42	1.24

36	ID	After being involved with people, I find out what they are really like	2.55	1.13

37	ID	People cannot guess how I'm going to behave	2.29	1.17

38	RT	When everything is confused, I feel that way inside	2.71	1.27

39	RT	I am not sure whether a voice I have heard is my imagination	1.67	1.00

40	RT	When I am confused, things in the outside world don't make sense either	2.30	1.26

41	RT	I feel as if I'm someone else	1.53	0.89

42	RT	I see things that turn out to be something else	1.57	0.90

43	RT	When uncomfortable, I can't tell whether it is emotional or physical	2.06	1.10

44	RT	I can see/hear things that nobody else can see/hear	1.35	0.76

45	RT	I hear things that are not really there	1.31	0.73

46	RT	I have heard or seen things without apparent reason	1.35	0.77

47	RT	I do things to upset other people	1.62	0.94

48	RT	I can't tell whether certain physical sensations are real	1.39	0.80

49	RT	My wishes/thoughts will come true as if by magic	1.54	0.87

50	RT	People see me as rude or inconsiderate	1.39	0.76

51	RT	I understand things that nobody else is able to understand	1.58	0.76

52	RT	I cannot tell when certain things would appear crazy to others	1.52	0.88

53	RT	I have seen things that do not exist	1.28	0.76

54	RT	I feel as if I have been somewhere before when I really haven't	2.32	1.16

55	RT	I can't tell whether I simply want something to be true	1.45	0.87

56	RT	Things will happen by thinking about them	1.38	0.75

57	RT	I never know how to conduct myself with people	1.66	1.01

58	AG	I enjoy seeing other people suffer	1.34	0.75

59	AG	When we disagreed about how to solve a problem, I couldn't stand it	1.48	0.91

60	AG	I have intentionally harmed someone	1.96	1.06

61	AG	To maintain control, you have to make people afraid of you	1.49	0.88

62	AG	I have seriously harmed someone in self-defence	1.91	1.09

63	AG	I control others by making them feel guilty	1.22	0.63

64	AG	I inflict physical harm on others	1.14	0.54

65	AG	I neglect my physical health	1.45	0.84

66	AG	You can obtain what you want by hurting yourself	1.26	0.77

67	AG	I like having others afraid of me	1.19	0.57

68	AG	I can't resist doing things which others consider hurtful but relieve tension	1.19	0.59

69	AG	The suffering of other people is exciting	1.19	0.59

70	AG	When people don't understand/mess things up I become hostile	2.02	1.15

71	AG	I enjoy making other people suffer	1.17	0.56

72	AG	It is a big relief to cause physical pain to myself	1.13	0.48

73	AG	I enjoy dangerous activities	1.23	0.60

74	AG	I have made an attempt at suicide	1.18	0.57

75	AG	I lose my patience and later regret it	1.76	1.02

76	MV	Everybody would steal if not afraid	1.79	1.14

77	MV	I feel justified in taking things that aren't mine if I can do so safely	1.31	0.74

78	MV	There are periods of time when I've acted in an immoral or amoral way	1.73	0.93

79	MV	People pretend to feel guilty when afraid of being caught	1.46	.077

80	MV	Everybody is out to get things for themselves	1.55	0.91

81	MV	One cannot judge others' real feelings from their surface behaviour	2.16	1.20

82	MV	Everybody pretends to be concerned about others and moral values	1.50	0.83

83	MV	I'm free of guilty feelings	1.46	0.90

The wordings of each item are abbreviated.

AG, Aggression; ID, Identity Diffusion; MV, Moral Value; PD, Primitive Defences; RT, Reality Testing.

We were interested in developing a Japanese version of the instrument that would resemble the original as closely as possible in terms of item content and factor structure, rather than constructing a new personality measure using all the IPO items. We therefore set the number of factors at three for the primary items and two for the additional items as suggested by the original authors. If we identified IPO items that loaded most highly on a factor other than the one that would have been expected from the original theory, we excluded them from the subsequent factor analyses (for example, if an item that was originally categorised as belonging to Reality Testing showed higher factor loading on the Identity Diffusion factor, we excluded it from the analysis). We also excluded IPO items with factor loading of less than 0.45 from the subsequent factor analysis. Thus, in the final factor analyses each factor contained a reduced number of items that belonged to the same category as defined in the original study [8].

In order to confirm the stability of the factor structures obtained from the above exploratory factor analyses, we performed a series of confirmatory factor analyses separately for the primary and additional items using another randomly generated subset of students. The fit of each model with the data was examined in terms of χ^2 ^(CMIN), goodness-of-fit index (GFI), adjusted goodness-of-fit index (AGFI), comparative fit index (CFI), and root mean square error of approximation (RMSEA). According to conventional criteria, a good fit would be indicated by CMIN/df <2, GFI >0.95, AGFI >0.90, CFI >0.97, and RMSEA <0.05; an acceptable fit by CMIN/df <3, GFI >0.90, AGFI >0.85, CFI >0.95, and RMSEA <0.08 [10]. The Akaike Information Criterion (AIC) was used to compare different models; a model with an AIC at least 2 points lower is regarded as a better model.

All the statistical analyses were conducted using SPSS version 14.0 (SPSS, Chicago, IL, USA) and Amos version 6.0 (SPSS).

### Results

#### Basic statistics

Means and SDs of all the IPO items among all students are presented in Table 1. The mean of 20 items was less than 1.4. The score of 1 ('never true') was reported by 75.1% to 92.1% of the participants for such items. Thus, they were excluded from subsequent factor analyses.

#### Factor structure

We performed an exploratory factor analysis on all items originally categorised as primary, using a randomly selected subset of students (N = 353). This showed that (1) almost all items with high factor loadings on the first factor were those originally categorised as Identity Diffusion; (2) all items with high factor loadings on the second factor were those originally categorised as Reality Testing; (3) almost all items with high factor loadings on the third factor were those originally categorised as Primitive Defence (Table 2). However, 18 items showed no loading of up to 0.45 or more for all three factors, and we therefore excluded these items from the subsequent analyses. Items 38 (originally a Reality Testing item), 17 (originally an Identity Diffusion item), and 22 (originally an Identity Diffusion item) showed a factor loading of 0.45 or more but these were found not to belong to the factor of their original category. Thus, we also excluded these items from the subsequent analyses.

**Table 2:** Exploratory factor analysis of the primary Inventory of Personality Organisation (IPO) items (N = 353)

No.	Question	Factor		
		
		1	2	3
29	Important people suddenly change towards me	0.80	-0.12	-0.05

38	When everything is confused, I feel that way inside	0.73	0.00	-0.06

28	When others see me as having succeeded, I'm elated	0.64	-0.07	0.05

36	After being involved with people, I find out what they are really like	0.63	0.00	0.07

30	It is hard for me to be sure about what others think of me	0.62	-0.28	0.24

35	My goals keep changing	0.54	0.38	-0.30

34	My life goals change frequently	0.52	0.38	-0.33

27	I pick up interests and then drop them	0.50	0.21	-0.18

31	Being alone is difficult	0.48	-0.06	-0.22

20	I behave differently in different situations	0.47	-0.22	0.41

32	I see myself in different ways at different times	0.46	0.17	0.10

25	It's hard for me to say no	0.46	0.04	0.03

21	I fluctuate between being warm and cold	0.43	-0.14	0.42

14	I do things that I later find hard to believe I did	0.40	0.13	0.19

33	In an intimate relationship, I'm afraid of losing a sense of myself	0.40	0.36	-0.10

40	When I am confused, things in the outside world don't make sense either	0.40	0.31	0.05

5	I admire people in order to feel secure	0.37	-0.19	0.33

24	I do things on impulse that are socially acceptable	0.32	0.05	0.29

37	People cannot guess how I'm going to behave	0.32	0.14	0.22

6	I do things that at other times I think are unwise	0.32	0.09	0.07

26	My life seems like a series of short stories	0.32	0.22	0.02

19	My tastes and opinions are borrowed from other people	0.29	0.15	0.21

13	I have favourite people whom I idealise	0.21	-0.02	0.12

55	I can't tell whether I simply want something to be true	-0.04	0.85	-0.04

42	I see things that turn out to be something else	-0.01	0.73	0.00

51	I understand things that nobody else is able to understand	-0.08	0.68	0.10

39	I am not sure whether a voice I have heard is my imagination	0.07	0.63	-0.01

54	I feel as if I have been somewhere before when I really haven't	-0.02	0.61	-0.04

43	When uncomfortable, I can't tell whether it is emotional or physical	0.13	0.55	0.01

41	I feel as if I'm someone else	0.06	0.54	0.12

47	I do things to upset other people	-0.08	0.53	0.14

52	I cannot tell when certain things would appear crazy to others	-0.04	0.51	0.21

49	My wishes/thoughts will come true as if by magic	-0.15	0.49	0.08

57	I never know how to conduct myself with people	0.13	0.46	0.19

4	People turn against me or betray me	-0.12	-0.05	0.69

11	People use me	-0.03	-0.07	0.64

15	People either overwhelm me with love or abandon me	-0.17	0.27	0.61

10	People are basically either good or bad	-0.32	0.15	0.58

17	Others see me as quite different from the way I really am	0.08	0.11	0.53

12	I act in unpredictable and erratic ways	0.13	0.01	0.52

22	I provoke people to get my way	-0.11	0.31	0.51

8	I don't get what I want	0.13	0.02	0.46

16	I feel things with either joy or despair	0.17	0.15	0.44

18	I'm different at home than at work/school	0.33	-0.01	0.40

3	It has been a long time since anyone taught me anything I did not know	-0.14	0.26	0.39

9	I behave in contradictory ways	0.04	0.32	0.36

1	I am a 'hero worshiper'	-0.02	0.04	0.34

2	People I once thought highly of have disappointed me	0.19	0.06	0.33

23	I can't explain changes in my behaviour	0.17	0.07	0.33

	Percentage variance explained	28.6%	4.5%	3.5%

We then factor analysed the remaining 28 primary items (Table 3). All the items with high factor loadings on the first factor belonged to Reality Testing, all the items with high factor loadings on the second factor belonged to Identity Diffusion, and all the items with high factor loadings on the third factor belonged to Primitive Defence. However, four items (items 8, 12, 31, and 32) showed a factor loading of less than 0.45 and we therefore dropped from the final subscale construction. Our dataset ultimately consisted of 11 items for Reality Testing, 9 items for Identity Diffusion, and 4 items for Primitive Defence.

**Table 3:** Revised exploratory factor analysis of the primary Inventory of Personality Organisation (IPO) items (N = 353)

No.	Question	Factor		
		
		1	2	3
55	I can't tell whether I simply want something to be true	0.85	-0.10	0.01

42	I see things that turn out to be something else	0.75	-0.06	0.02

51	I understand things that nobody else is able to understand	0.73	-0.08	0.06

54	I feel as if I have been somewhere before when I really haven't	0.64	-0.08	-0.01

39	I am not sure whether a voice I have heard is my imagination	0.63	0.02	0.04

49	My wishes/thoughts will come true as if by magic	0.59	-0.17	-0.03

47	I do things to upset other people	0.57	-0.03	0.06

52	I cannot tell when certain things would appear crazy to others	0.56	0.01	0.11

41	I feel as if I'm someone else	0.54	0.04	0.16

43	When uncomfortable, I can't tell whether it is emotional or physical	0.54	0.10	0.06

57	I never know how to conduct myself with people	0.48	0.17	0.15

29	Important people suddenly change towards me	-0.11	0.78	0.01

28	When others see me as having succeeded, I'm elated	-0.04	0.70	-0.02

30	It is hard for me to be sure about what others think of me	-0.24	0.69	0.22

36	After being involved with people, I find out what they are really like	0.03	0.64	0.08

20	I behave differently in different situations	-0.11	0.56	0.30

34	My life goals change frequently	0.38	0.50	-0.37

35	My goals keep changing	0.40	0.49	-0.30

25	It's hard for me to say no	0.04	0.45	0.08

27	I pick up interests and then drop them	0.18	0.45	-0.10

32	I see myself in different ways at different times	0.20	0.45	0.12

31	Being alone is difficult	-0.18	0.43	-0.05

11	People use me	-0.05	0.07	0.71

4	People turn against me or betray me	-0.02	0.02	0.67

10	People are basically either good or bad	0.18	-0.22	0.57

15	People either overwhelm me with love or abandon me	0.33	-0.04	0.51

12	I act in unpredictable and erratic ways	0.12	0.19	0.40

8	I don't get what I want	0.07	0.29	0.36

	Percentage variance explained	29.4%	6.7%	5.3%

**Figure 1:**
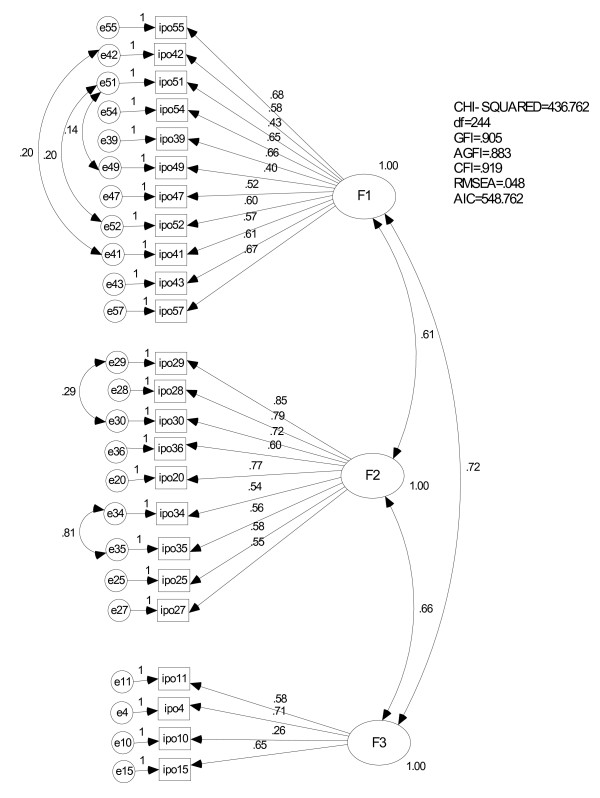
**Confirmatory factor analysis of the primary Inventory of Personality Organisation (IPO) items**. The IPO numbers correspond to those appearing in Table 1.

We then performed confirmatory factor analyses (CFA) of the final 24 primary items using the other group of students (N = 348). In the initial model we posited covariances between all three factors; this barely failed to reach an acceptable level of significance: χ^2^/df = 2.9, GFI = 0.845, AGFI = 0.813, CFI = 0.796, RMSEA = 0.075, AIC = 836.0. Taking into account the greatest modification index of covariance, we developed a revised model (Figure 1) that fit the data better: χ^2^/df = 1.8, GFI = 0.905, AGFI = 0.883, CFI = 0.919, RMSEA = 0.048, AIC = 548.8.

Similarly we performed an EFA of the additional items of the IPO. It is of note that the original concept included under the rubric of 'additional items' three items (items 6, 14, and 17) that were also categorised as primary items. Almost all the items with high factor loadings on the first factor were those originally categorised as Aggression. Almost all the items with high factor loadings on the second factor were those originally categorised as Moral Value (Table 4). However, two items showed no factor loading of up to 0.45 or more for both factors. In addition, items 14 and 17 (originally Moral Value items) showed a factor loading of 0.45 or more on the first factor. These factors were excluded before repeating the EFA. The revised EFA yielded two factors with six and seven items, respectively (Table 5).

**Table 4:** Exploratory factor analysis of the additional Inventory of Personality Organisation (IPO) items (N = 353)

No.	Question	Factor	
		
		1	2
59	When we disagreed about how to solve a problem, I couldn't stand it	0.84	-0.16

62	I have seriously harmed someone in self-defence	0.79	-0.11

60	I have intentionally harmed someone	0.75	0.01

61	To maintain control, you have to make people afraid of you	0.64	0.11

14	I do things that I later find hard to believe I did	0.62	-0.14

17	Others see me as quite different from the way I really am	0.62	0.00

75	I lose my patience and later regret it	0.55	0.20

70	When people don't understand/mess things up I become hostile	0.47	0.33

6	I do things that at other times I think are not too wise	0.27	0.14

80	Everybody is out to get things for themselves	-0.21	0.85

79	People pretend to feel guilty when afraid of being caught	-0.17	0.79

81	One cannot judge others' real feelings from their surface behaviour	0.09	0.63

76	Everybody would steal if not afraid	0.04	0.60

82	Everybody pretends to be concerned about others and moral values	0.22	0.58

78	There are periods of time when I've acted in an immoral or amoral way	0.28	0.51

83	I'm free of guilty feelings	-0.06	0.50

65	I neglect my physical health	0.21	0.29

	Percentage variance explained	35.2%	8.0%

**Table 5:** Revised exploratory factor analysis of the additional Inventory of Personality Organisation (IPO) items (N = 353)

No.	Question	Factor	
		
		1	2
59	When we disagreed about how to solve a problem, I couldn't stand it	0.90	-0.22

62	I have seriously harmed someone in self-defence	0.87	-0.17

60	I have intentionally harmed someone	0.73	0.02

61	To maintain control, you have to make people afraid of you	0.68	0.09

75	I lose my patience and later regret it	0.54	0.21

70	When people don't understand/mess things up I become hostile	0.49	0.33

80	Everybody is out to get things for themselves	-0.16	0.82

79	People pretend to feel guilty when afraid of being caught	-0.18	0.81

81	One cannot judge others' real feelings from their surface behaviour	0.12	0.62

82	Everybody pretends to be concerned about others and moral values	0.24	0.58

76	Everybody would steal if not afraid	0.11	0.55

83	I'm free of guilty feelings	-0.07	0.52

78	There are periods of time when I've acted in an immoral or amoral way	0.33	0.47

	Percentage variance explained	40.4%	9.9%

**Figure 2:**
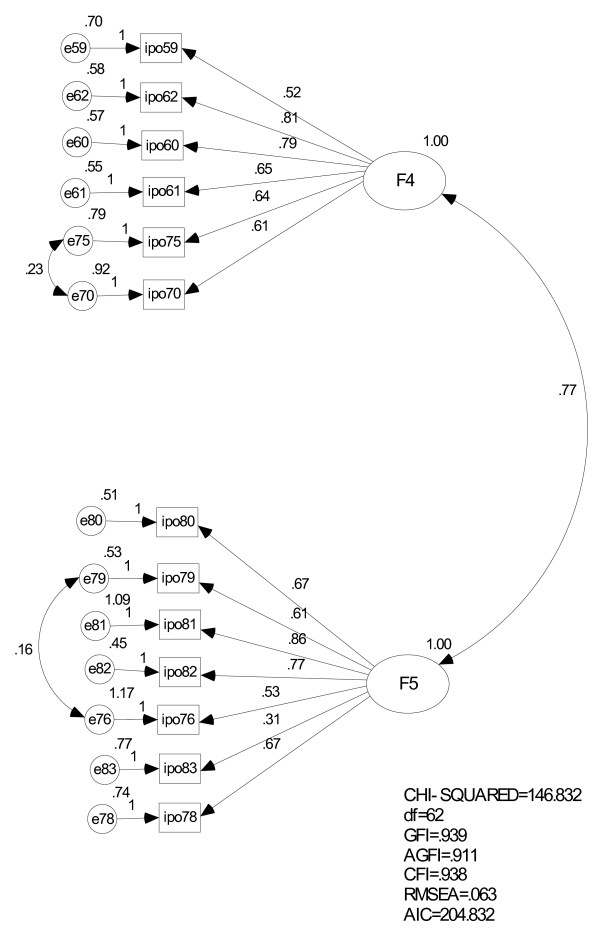
**Confirmatory factor analysis of the additional Inventory of Personality Organisation (IPO) items**. The IPO numbers correspond to those appearing in Table 2.

As in the primary items, we performed a CFA on these 13 items using the second random group of students. The initial model posited a covariance between the 2 factors, with a relatively poor fit of the data: χ^2^/df = 2.8, GFI = 0.925, AGFI = 0.863, CFI = 0.916, RMSEA = 0.072, AIC = 233.8. Taking into account the greatest modification index of covariance we created a revised model (Figure 2) that fit the data better: χ^2^/df = 2.4, GFI = 0.939, AGFI = 0.911, CFI = 0.938, RMSEA = 0.063, AIC = 204.8.

### Discussion

This study supports the validity of the Japanese version of the IPO given to Japanese student populations. The translation accuracy of a self-report measure for psychological traits or states does not necessarily guarantee the transferability of its contents and factor structure, which may be dependent on the cultural and linguistic background of the population in which it was developed [11]. It is believed that this is particularly the case in the assessment of personality traits and personality disorders. Thus, it is encouraging that the Japanese version of the IPO yielded a three-factor structure for primary items and a two-factor structure for additional items, in agreement with Kernberg's theoretical considerations. The confirmatory factor analysis further supported the fit of the model to our data.

A drawback of study 1 is that of 83 items of the IPO, only 24 primary items and 13 secondary items were retained as usable in the final Japanese version. This is due to our aim to yield the same factor structure as that in the original scale. As its results, however, 46 (55%) items were dropped. To start with, 20 items showed a low base rate. For example, items such as 'I can see/hear things that nobody else can see/hear', 'I hear things that are not really there', and 'I have heard or seen things without apparent reason' represent psychosis-like experiences and may be rare among undergraduate students, items such as 'I enjoy seeing other people suffer', 'I control others by making them feel guilty', and 'I inflict physical harm on others' represent aggressive tendencies and items such as 'I feel justified in taking things that aren't mine if I can do so safely' represent antisocial behaviours. Therefore these items with a low base rate may be rare among non-clinical population.

The item 'When everything is confused, I feel that way inside', which was originally a Reality Testing item, showed higher factor loading for the first factor in this study, which was interpreted as reflecting Identity Diffusion. Unlike the other Reality Testing items that describe a failure to differentiate self from non-self in the realms of thought and perception, as occurs with ideas of reference, depersonalisation, and illusion, this item refers to intrapsychiatric confusion resonating to the outer world. Using the original IPO, Lenzenweger *et al*. [8] performed a confirmatory factor analysis of the items in the three primary subscales. In their three-factor model, the item 'When everything is confused, I feel that way inside' demonstrated the lowest loading for the Reality Testing factor. In retrospect, we therefore consider that this item has a much stronger component of Identity Diffusion than of Reality Testing.

The present study employed only students. They tended to be young and may be well functioning and different from a population of patients with personality pathology in terms of the factor structure. A patient population may contain more people with higher base rates of the above-mentioned items. Hence a different factor structure may emerge. This issue should be studied in future works including clinical as well as non-clinical populations. Our study does not prove a final model of the factor structure of the Japanese version of the scale. Nevertheless, for the time being, we think the present factor structure may provide a tentative means to study the validity of the borderline personality concept at least among student populations using a self-report instrument.

## Study 2

### Methods

Study 1 demonstrated that the Japanese version of the IPO has a similar factor structure to the original version and that it is replicable in a randomly separated subgroup of the initial participant population. Therefore, we constructed the subscales of the Japanese IPO by adding the scores of items that belonged to each factor, and in doing so obtained five subscales.

As a study of the scale's concurrent validity we then compared the scores of the five subscales with those of other personality pathologies. We expected that the scores of the Japanese IPO would correspond substantially to the scores of the measures for the DSM borderline and narcissistic personality disorders in a psychiatric outpatient population. As a study of the scale's construct validity, we compared the scores of the subscales of the IPO with mood measurements, psychological well-being, adult attachment style, and early life experiences in outpatient and student populations. This is because borderline personality organisation or borderline personality disorders have often been linked to comorbid mood and anxiety disorders [12,13], dysfunctional attachment behaviours [14], and childhood experiences of childhood abuse [15-19], emotional neglect [19,20], and overprotection [20].

As a study of the scale's predictive validity, we prospectively monitored some students for depressive mood and suicidal ideation for 1 week, as borderline personality has been linked to self-harm [21].

#### Participants

We recruited university students used for study 1 and psychiatric outpatients. For the sake of brevity, different questionnaires were used for different students. The first subset of students (N = 271) was given the Hospital Anxiety and Depression Scale (HADS) [22] and the Inventory of Psychological Well-Being (PWB) [23], in addition to the IPO, on a single occasion. The second subset of students (N = 430) had been participating in a longitudinal weekly follow-up study on mental health of university students that consisted of 9 waves. On different occasions the students were distributed sets of questionnaires, including the IPO (wave 7, N = 430), the Adult Attachment Relationship Questionnaire (AARQ) [24] (wave 5), the Parental Bonding Instrument (PBI) [25] (wave 3), the Child Abuse and Trauma Scale (CATS) [26] (wave 2), and the Suicidal Ideation item from the Self-Rating Depression Scale (SDS) [27] (each wave). The numbers of students used as samples for different questionnaires will be noted.

The outpatient group consisted of 177 individuals attending a psychiatric clinic over the course of 2 months. Patients with dementia, mental retardation, and alcohol or drug abuse were not solicited for participation in the survey. However, full IPO data were available only from 138 patients; the data of these patients were used for further analyses. The mean (SD) age of this group was 52.8 (16.7) years, and the group was comprised of 54 men and 82 women; 2 patients did not report their gender. Women (mean = 55.4, SD = 16.5) were slightly but significantly (t = 2.13, *P *< 0.05) older than men (mean = 49.4, SD = 15.7). The mean (SD) duration of the current condition was 87.7 (86.7) months. Only 18% of patients reported that their condition had started within 1 year prior to the survey; 53% of them reported that their condition had started more than 5 years prior. The mean (SD) duration of the attendance at this clinic was 59.0 (58.5) months. Only 29% of patients stated that they had begun attending the clinic within 1 year prior to the survey and 43% of them reported attending the clinic for longer than 5 years. Because of the anonymity of the questionnaire we failed to identify the proportion of psychiatric diagnoses in the patient population.

#### Measurement

Personality organisation: the IPO was used for assessment. Composite variables were constructed as new subscales of the inventory based on the factor analyses described in study 1.

##### DSM-III-R personality disorders

The Personality Diagnostic Questionnaire-Revised (PDQ-R) [28] is a self-report measure that identifies each of the personality disorders listed in the DSM-III-R. Each of the DSM-III-R personality disorder categories is assessed according to an algorithm derived from items on a 2-point scale (true or false) corresponding to the items of the diagnostic criteria. The psychometric properties of the PDQ-R and its predecessor, the PDQ, have been reported and verified [29-34]. The PDQ-R was translated into Japanese by Dr T Nagata, Department of Neuropsychiatry, Osaka City University Medical School, Osaka, Japan, and the wording was verified by back-translation. In this study, we used only the sections for borderline and narcissistic personality disorders. Rather than using the categorical assessment of personality diagnosis, we used the total number of items selected by each person. The range of total scores is from 0 to 8 for borderline personality disorder and from 0 to 9 for narcissistic personality disorder. A higher score indicates more severe pathology of each disorder.

##### Depression and anxiety

The HADS [22], Japanese version [35], measures the cognitive symptoms of depression and anxiety. It consists of 14 items, and the depression (HAD-D) and anxiety (HAD-A) subscales each include 7 items on a 4-point scale ranging from 0 (low depression or anxiety) to 3 (high depression or anxiety). The total score of the HAD-D/HAD-A can range from 0 to 21, and higher scores indicate more severe depression or anxiety. Missing data among cases were substituted with mean values unless data for more than two items were missing.

##### Psychological well-being

The PWB [23] is a measure of the postulated 6 dimensions of psychological well-being, and includes 84 items on a 2-point scale ('Yes' = 1 and 'No' = 0). The six subscales were derived from literature review and theoretical considerations. The six scales are (1) self-acceptance (possessing a positive attitude towards oneself), (2) positive relationships with others (having warm, satisfying, trusting relationships with others), (3) autonomy (self-determination and independence), (4) environmental mastery (a sense of mastery and competence in handling one's environment), (5) purpose in life (having goals for one's life and a sense of direction), and (6) personal growth (a feeling of continued development). Each subscale consists of 14 items, and the scores for each subscale range from 0 to 14. Missing data were substituted with mean values unless data for more than four items were missing. This inventory was translated into Japanese [36] with permission of the original author. The psychometric properties of the original version of the inventory have been reported for both the original [37-39] and the Japanese versions [36].

##### Adult attachment style

The Relationship Questionnaire (RQ) [24] measures four categories of adult attachment: Secure, Fearful, Preoccupied, and Dismissing. The last three categories were grouped as insecure attachment styles. The RQ is composed of four paragraphs, describing each attachment style. Participants were asked to rate the extent to which each description corresponded to their relationship with their partner. If they had no definite partner, they were requested to imagine an intimately related person of the opposite gender when answering the question. The reliability [24] and validity [40] of the RQ have been reported. Participants replied using a 7-point scale (1 = 'Does not apply to me at all' to 7 = 'Applies to me very much'). The psychometric properties have been reported in married couples, romantic partners and undergraduate students in Canada [40,41]. With the permission of Dr. Kim Bartholomew, Department of Psychology, Simon Fraser University, 8888 University Drive, Burnaby, British Columbia, Canada V5A 1S6, the RQ was translated into Japanese (TK). In accordance with Tanaka *et al*. [42] the Total Attachment Score (TAS) was calculated by subtracting the three insecure attachment item scores (fearful, preoccupied, and dismissing) from the secure item score. A higher score would then demonstrate a more secure attachment style.

##### Self-efficacy

Self-efficacy was measured using the Self-Efficacy Scale (SES) [43,44]. Efficacy beliefs regulate stress and anxiety through their impact on coping behaviour [45,46]. This is noted to determine a person's coping effort and persistence [47]. The SES was translated by Narita *et al*. with the original author's permission, and its reliability and validity were subsequently examined [48]. The Japanese version of the SES is comprised of 23 items with a 5-point scale from 0 to 4. A higher score indicates better self-efficacy.

##### Early life experiences

We evaluated these using the Parental Bonding Instrument (PBI) [25] and the CATS [26]. The PBI is a self-report questionnaire that is intended to retrospectively assess parental attitudes toward the subject as a child. The 25 items were scored on a 4-point scale (0 = very unlikely, 3 = very likely). There are 2 subcategories: Care (12 items) and Overprotection (13 items). Higher scores reflect a higher Care or higher Overprotection experience. Good reliability has been reported for the PBI [25]. Kitamura and Suzuki [49,50] have translated the PBI into Japanese, using back-translation into English to verify the wording. The validity of the instrument has been confirmed by the high agreement between PBI scores of mother and father, respectively, recorded by the students, father, and mother. Mean values were substituted for missing items when at least 20 out of the 25 items were answered.

The CATS is a self-report measure of the experiences of sexual abuse, neglect, and punishment (physical abuse). It consists of 38 items on a 5-point scale (0 = never, 4 = always). It has 3 subcategories: Sexual Abuse (6 items), Neglect (14 items), and Punishment (6 items). Mean values were substituted for missing items when at least 31 out of the 38 items were answered.

##### Suicidal ideation during prospective follow-up

Current suicidal ideation was rated on a 4-point scale (0 = never, 3 = almost always) by a single item reflecting suicidality in the SDS [27]. This is 'I feel that others would be better off if I were dead'. Because the score of this item was positively skewed (skewness = 2.61 and 2.62 for the first and third times, respectively), we log-transformed the score so that their skewness became slightly lower (2.13 and 2.16, respectively).

#### Procedure

All the questionnaires were anonymously distributed and collected in a university class. This project was approved by the Ethical Committee of Kumamoto University Graduate School of Medical Sciences.

#### Statistical analysis

First, we associated the scores of all five subscales of the Japanese IPO with demographic variables of all the students and outpatients. Then we correlated the IPO scores with those of the PDQ-R, as well as with those of the HADS, PWB, RQ, SES, PBI, CATS and the Suicidal Ideation item of the SDS. These correlations were controlled for the effects of age and gender (partial correlations). Finally, in the second subset of students, the time 2 (wave 8) Suicidal Ideation score was regressed on the predictor variables. The predictor variables were forced to enter in the following order: (1) age and gender, (2) Suicidal Ideation score at time 1 (wave 7), and (3) the time 1 (wave 7) IPO subscales.

All the statistical analyses were conducted using SPSS version 14.0.

## Results

### IPO subscales and demographic variables in patient and student populations

Means and SDs of all subscales of the Japanese version of the IPO, as well as demographic variables (age and gender) of all students and outpatients, are presented in Table 6 (age and gender were unknown for one student and two outpatients). All the IPO subscale scores were moderately correlated with each other. They decreased with increasing participant age. t Tests revealed that, as compared to women, men scored significantly higher in Primitive Defences (men: mean = 7.6, SD = 2.8; women: mean = 7.1, SD = 3.0; *P *< 0.05) and Moral Value (men: mean = 12.8, SD = 5.3; women: mean = 11.5, SD = 4.6; *P *< 0.01) but significantly lower in Identity Diffusion (men: mean = 22.4, SD = 7.6; women: mean = 23.6, SD = 7.0; *P *< 0.05).

**Table 6:** Means and standard deviations (SDs) of and correlations between the Inventory of Personality Organisation (IPO) subscale scores and demographic variables (N = 839)

	1	2	3	4	5	6	7	8
1: Age	-							

2: Gender (men 1: women 2)	-0.09*	-						

3: group (students 1: outpatients 2)	0.87***	-0.13***	-					

4: Primitive Defences	0.02	-0.08*	0.06	-				

5: Identity Diffusion	-0.29***	0.07*	-0.22***	0.42***	-			

6: Reality Testing	-0.15***	-0.03	-0.10**	0.49***	0.55***	-		

7: Aggression	-0.14***	-0.04	-0.10**	0.45***	0.55***	0.65***	-	

8: Moral Value	-0.12**	-0.12**	-0.05	0.46***	0.47***	0.57***	0.65***	-

Possible range of score	-	-	-	4 to 20	9 to 45	11 to 55	6 to 30	7 to 35

Mean	25.0	1.7	1.2	7.2	23.3	18.6	10.5	11.9

SD	14.2	0.4	3.7	2.9	7.2	7.0	4.5	4.8

*P < 0.05; **P < 0.01; ***P < 0.001.

Three IPO subscales, Identity Diffusion, Reality Testing and Aggression, showed higher scores among students than outpatients. However, these findings may be due to confounding because (1) all the IPO subscale scores were negatively correlated with age and (2) outpatients (mean = 52.8, SD = 16.7) were significantly (t = 23.3, *P *< 0.000) older than students (mean = 19.6, SD = 2.3). Thus partial correlations were performed for each of the IPO subscale scores with the group (students 1: outpatients 2) controlled for age and gender. After controlling for age and gender, the IPO subscale scores were positively correlated with group (Primitive Defences *r *= 0.07, *P *= 0.026; Identity Diffusion *r *= 0.06, *P *= 0.082; Reality Testing *r *= 0.07, *P *= 0.058; Aggression *r *= 0.06, *P *= 0.105; Moral Value *r *= 0.11, *P *= 0.002).

### IPO subscales and personality disorder diagnoses

The PDQ-R was administered to 138 outpatients. As expected, all IPO subscales were correlated with the Narcissistic Personality and Borderline Personality scores (Table 7). Narcissistic Personality scores decreased with increasing participant age. The above findings were virtually the same even after controlling for age and gender (one and two cases were missing for age and gender, respectively).

**Table 7:** The Inventory of Personality Organisation (IPO) subscales and Personality Diagnostic Questionnaire-Revised (PDQ-R) subscales among outpatients (N = 138)

	Narcissistic personality	Borderline personality
Mean (standard deviation)	2.4 (1.6)	2.0 (1.6)

Primitive Defences	0.37*** (0.36***)	0.37*** (0.35***)

Identity Diffusion	0.55*** (0.48***)	0.54*** (0.51***)

Reality Testing	0.55*** (0.51***)	0.57*** (0.53***)

Aggression	0.57*** (0.51***)	0.58*** (0.55***)

Moral Value	0.53*** (0.46***)	0.45*** (0.41***)

Age	-0.37***	0.22*

Gender (men 1: women 2)^a^	-0.05	0.04

^a^Gender was unknown for one case.

*P < 0.05; **P < 0.01; ***P < 0.001; parentheses indicate partial correlations controlling for age and gender.

### IPO subscales and dysphoric mood

The HADS was distributed to all the outpatients (N = 138) and the first subset of students (N = 271). As expected, all IPO subscales were significantly correlated with each of the HADS subscales (Table 8). HAD-D was correlated negatively with age whereas HAD-A was correlated positively with age. HAD-A was slightly but significantly higher among the outpatients (mean = 6.4, SD = 4.2) than the students (mean = 5.1, SD = 3.2). The outpatients (mean = 7.0, SD = 3.9) and students (mean = 7.4, SD = 3.7) did not differ in HAD-D. The above findings were virtually the same even after controlling for age, gender, and group.

**Table 8:** The Inventory of Personality Organisation (IPO) subscales and the Hospital Anxiety and Depression Scale (HADS) among outpatients and a subset of students (N = 403)

	HAD-D (N = 403)	HAD-A (N = 403)
Mean (standard deviation)	7.3 (3.8)	5.5 (3.6)

Primitive Defences	0.36*** (0.38***)	0.42*** (0.43***)

Identity Diffusion	0.39*** (0.37***)	0.32*** (0.40***)

Reality Testing	0.48*** (0.47***)	0.38*** (0.42***)

Aggression	0.34*** (0.32***)	0.23*** (0.28***)

Moral Value	0.28*** (0.27***)	0.26*** (0.33***)

Age	-0.14**	0.13**

Gender (men 1: women 2)^a^	0.09	0.04

Group (students 1: outpatients 2)	-0.06	0.18***

^a^Gender was unknown for one case.

*P < 0.05; **P < 0.01; ***P < 0.001; parentheses indicate partial correlations controlling for age, gender, and group.

HAD-A, Hospital Anxiety and Depression Scale-Anxiety; HAD-D, Hospital Anxiety and Depression Scale-Depression.

### IPO subscales and psychological well-being

The PWB was distributed to the first subset of students (N = 271). As expected, all IPO subscales were significantly negatively correlated with each of the PWB subscales except for non-significant correlations of Autonomy with Primary Defences, Aggression, and Moral Values (Table 9). All PWB subscales increased with participant age. The above findings were virtually the same even after controlling for the age and gender.

**Table 9:** The Inventory of Personality Organisation (IPO) subscales and the Inventory of Psychological Well-Being (PWB) among a subset of students (N = 271)

	Autonomy (N = 264)	Environmental mastery (N = 264)	Personal growth (N = 264)	Positive relationship with others (N = 264)	Purpose in life (N = 264)	Self acceptance (N = 264)
Mean (standard deviation)	6.6 (3.3)	7.4 (2.7)	10.0 (3.0)	9.7 (2.9)	8.8 (3.4)	6.7 (3.3)

Primitive Defences	-0.04 (-0.05)	-0.23*** (-0.23***)	-0.22*** (-0.23***)	-0.40*** (-0.39***)	-0.25*** (-0.24***)	-0.30*** (-0.30***)

Identity Diffusion	-0.30*** (-0.27***)	-0.47*** (-0.45***)	-0.28*** (-0.25***)	-0.42*** (-0.41***)	-0.39*** (-0.36***)	-0.43*** (-0.41***)

Reality Testing	-0.17** (-0.16*)	-0.42*** (-0.40***)	-0.25*** (-0.23***)	-0.39*** (-0.38***)	-0.41*** (-0.39***)	-0.40*** (-0.38***)

Aggression	-0.02 (-0.02)	-0.38*** (-0.37***)	-0.20** (-0.20**)	-0.46*** (-0.46***)	-0.32*** (-0.30***)	-0.33*** (-0.32***)

Moral Value	0.02 (0.03)	-0.31*** (-0.29***)	0.24*** (-0.23***)	-0.44*** (-0.43***)	-0.39*** (-0.37***)	-0.30*** (-0.28***)

Age	0.19**	0.20***	0.24***	0.09	0.24***	0.23***

Gender (men 1: women 2)	-0.10	0.04	-0.04	0.10	0.10	0.02

*P < 0.05; **P < 0.01; ***P < 0.001; parentheses indicate partial correlations controlling for age and gender.

### IPO subscales and adult attachment style

The RQ was distributed to the second subset of students (N = 430). Usable RQ data were available from 369 students. As expected, students with lower (insecure) Adult Attachment were more likely to show higher scores for each of the IPO subscales (Table 10). This was also the case after controlling for age and gender.

**Table 10:** The Inventory of Personality Organisation (IPO) subscales and the Relationship Questionnaire (RQ) among a subset of students

	Adult Attachment (N = 369)	Self Efficacy (N = 371)
Mean (standard deviation)	-2.8 (4.2)	48.8 (12.4)

Primitive Defences	-0.44*** (-0.44***)	-0.24*** (-0.24***)

Identity Diffusion	-0.37*** (-0.37***)	-0.32*** (-0.31***)

Reality Testing	-0.30*** (-0.30***)	-0.20*** (-0.20***)

Aggression	-0.30*** (-0.30***)	-0.28*** (-0.28***)

Moral Value	-0.31*** (-0.31***)	-0.25*** (-0.24***)

Age	0.02	0.08

Gender (men 1: women 2)	-0.01	-0.06

*P < 0.05; **P < 0.01; ***P < 0.001; parentheses indicate partial correlations controlling for age and gender.

### IPO subscales and self-efficacy

The SES was distributed to the second subset of students (N = 430). Usable data were available from 371 students. As expected, students with lower Self Efficacy were more likely to show higher scores for each of the IPO subscales (Table 10). This was also the case after controlling for age and gender.

### IPO subscales and early life experiences

The PBI and CATS were distributed to the second subset of students (N = 430). Usable data were available from 374 for paternal Care, 372 for paternal Overprotection, 288 for maternal Care, 283 for maternal Overprotection, and 369 for the CATS. As expected, all IPO subscales correlated negatively with parental Care, and positively with parental Overprotection and the three types of child abuse (except for lack of correlation between Identity Diffusion and Child Sexual Abuse) (Table 11). These were virtually the same after controlling for age and sex.

**Table 11:** The Inventory of Personality Organisation (IPO) subscales and the Parental Bonding Instrument (PBI) and Child Abuse and Trauma Scale (CATS) among a subset of students

	Fathers' Care (N = 374)	Fathers' Overprotection (N = 372)	Mothers' Care (N = 288)	Mothers' Overprotection (N = 283)	Child Sexual Abuse (N = 369)	Neglect (N = 369)	Punishment (N = 369)
Mean (standard deviation)	25.8 (7.4)	9.8 (5.9)	29.6 (5.6)	10.1 (6.3)	0.28 (1.21)	10.1 (8.4)	7.5 (3.7)

Primitive Defences	-0.23*** (-0.30***)	0.16** (0.21**)	-0.26*** (-0.29***)	0.23*** (0.23***)	0.24*** (0.27***)	0.33*** (0.26***)	0.21*** (0.22***)

Identity Diffusion	-0.12* (-0.22**)	0.13* (0.22***)	-0.14* (-0.19**)	0.18** (0.17**)	0.01 (0.06)	0.25*** (0.31***)	0.11* (0.16*)

Reality Testing	-0.20*** (-0.28***)	0.19*** (0.30***)	-0.27*** (-0.29***)	0.28*** (0.29***)	0.22*** (0.31***)	0.36*** (0.41***)	0.31*** (0.33***)

Aggression	-0.20*** (-0.28***)	0.13* (0.18**)	-0.23*** (-0.26***)	0.18** (0.19**)	0.17** (0.287***)	0.31*** (0.39***)	0.21*** (0.24***)

Moral Value	-0.12* (-0.17**)	0.14** (0.19**)	-0.19** (-0.22***)	0.22*** (0.23***)	0.19*** (0.32***)	0.30*** (0.37***)	0.17** (0.22***)

Age	-0.05	-0.03	-0.08	0.01	-0.04	0.05	0.08

Gender (men 1: women 2)	0.07	0.09	0.05	0.03	-0.10	0.09	0.01

The wordings of each item are abbreviated.

AG, Aggression; ID, Identity Diffusion; MV, Moral Value; PD, Primitive Defences; RT, Reality Testing.

### Prediction of suicidal ideation from IPO subscales

We correlated data on Suicidal Ideation, age, gender, and all IPO subscales obtained on time 1 (wave 7) with that obtained on time 2 (wave 8) (Table 12). One student did not report on Suicidal Ideation on time 1 (wave 7). A total of 61 students were not present on time 2 (wave 8) and thus no data could be gathered from them.

**Table 12:** Correlations of Suicidal Ideation, age, gender, and all Inventory of Personality Organisation (IPO) subscales in a subset of students (N = 430)

	1	2	3	4	5	6	7	8	9
1: Primitive Defences	-								

2: Identity Diffusion	0.47***	-							

3: Reality Testing	0.55***	0.53***	-						

4: Aggression	0.52***	0.52***	0.64***	-					

5: Moral Value	0.48***	0.40***	0.59***	0.64***	-				

6: Suicidal ideation T1	0.48***	0.29***	0.43***	0.36***	0.35***	-			

7: Suicidal ideation T2^a^	0.45***	0.23***	0.42***	0.28***	0.34***	0.75***	-		

8: Age	-0.07	-0.12*	-0.12*	-0.08	-0.09	-0.07	-0.05	-	

9: Gender	-0.04	0.14**	-0.06	-0.04	-0.06	0.02	0.02	-0.16**	-

^a^Data were not available from 61 students.

*P < 0.05; **P < 0.01; ***P < 0.001.

Time 1 (wave 7) Suicidal Ideation was significantly correlated with time 2 (wave 8) Suicidal Ideation. These two were correlated significantly with each of the IPO subscales that were measured at time 1 (wave 7). Age was slightly negatively correlated with Identity Diffusion and Reality Testing. Female gender was associated with Identity Diffusion.

When time 2 (wave 8) Suicidal Ideation was regressed on (1) age and gender, (2) time 1 (wave 7) Suicidal Ideation, and (3) all the IPO subscales, time 2 (wave 8) Suicidal Ideation was predicted significantly by time 1 (wave 7) Suicidal Ideation (Table 13). After controlling for the effects of age, gender, and wave 7 Suicidal Ideation, wave 8 Suicidal Ideation was still predictable by the IPO subscale scores. This was statistically significant for Reality Testing (standardised β = 0.105, *P *< 05). When wave 8 Suicidal Ideation was further regressed on the interaction terms of wave 7 Suicidal Ideation with each of the IPO subcategories, these did not prove to be significant in predicting the wave 8 Suicidal Ideation (data not shown).

**Table 13:** Regression analysis of time 2 (wave 8) Suicidal Ideation in a subset of students (N = 369)

	R2	Increase R2	F (*df*)	*P*****value	Standardised β	*P *value
First block	0.003	0.003	00.6 (2,365)	0.564		

Age					-0.006	0.871

Gender					-0.001	0.969

Second block	0.561	0.558	463.4 (1,364)	0.000		

Suicidal ideation T1					0.684	0.000

Third block	0.577	0.016	2.7 (5,359)	0.023		

Primitive Defences					0.063	0.160

Identity Diffusion					-0.062	0.159

Reality Testing					0.105	0.039

Aggression					-0.049	0.328

Moral Value					0.063	0.195

Adjusted R^2^	0.568					

*P < 0.05; **P < 0.01; ***P < 0.001.

## Discussion

The new subscale scores (other than Primitive Defences) derived from the confirmatory factor analysis in study 1 were higher among younger people. This may reflect underdeveloped personality maturation in adolescents and young adults. Because age may correlate with personality maturation, as suggested by the correlation studies, a population more diverse in terms of age is needed for future studies.

We expected that the IPO subscale scores would be higher among outpatients than among university students. This proved not to be the case. However, there is a great discrepancy in ages between the student and outpatient groups. The finding that the IPO subscale scores were all positively associated with outpatient status when controlling for the effects of age and gender suggest that the above-mentioned negative correlations were spurious, being confounded by the outpatients' greater average age. The lack of a clear difference between the student and outpatient populations may be due to the fact that only a small portion of the patients were in an acute phase of their illness, and most of them had been attending the clinic for years. In order to guarantee the anonymity of the participants' responses, we had no means of investigating the effects of psychiatric diagnosis on the IPO scores. It remains possible that the personality organisation measured by the IPO will be observed in people with other personality disorders, such as schizotypal, antisocial and histrionic. Future investigations should include patients in an acute phase with a definite diagnostic classification.

One indication of the concurrent validity of the IPO was its concordance with the severity of personality pathologies such as borderline and narcissistic personality disorders. The scores of the PDQ-R correlated significantly with all five IPO subscales, suggesting that the IPO reflects personality pathology in accordance with the DSM framework. However, it remains to be determined whether the IPO reflects personality pathology in general, or rather specific categories of DSM personality disorders. Another drawback of this investigation lies in the use of self-report as a measurement of DSM personality disorders [51]. Informants other than participants themselves should be included in future studies [52].

Another indication of the IPO's validity was its concordance with poor psychological adjustment. To gauge the presence of this state, we examined dysphoric mood (depression and anxiety), psychological well-being, adult attachment style, and self efficacy. We hypothesised that negative affects such as depression and anxiety would be stronger in those with more severe borderline personality pathology, and this hypothesis was supported by our results. Similarly, all measures of psychological well-being except Autonomy correlated negatively with the five subcategories of the IPO. Only Identity Diffusion and Reality Testing were characterised by poor Autonomy. Adult Attachment styles and Self Efficacy scores were negatively correlated with all the IPO subcategories. All of these data show that the Japanese version of the IPO resonates with Kernberg's theory, and that each of the subscales was associated with theory-driven measures of the constructs. We therefore hope that it may be useful in both clinical and non-clinical settings as a measure of borderline personality pathology.

To assess predictive validity, we examined whether IPO scores at one point would predict the suicidal ideation in a week's time. After controlling the effects of age and gender as well as of concurrent suicidality, a group of IPO subscales and particularly Reality Testing predicted an increase in suicidality in 1 week's time.

A drawback of this study is its reliance on a student population. Comorbid personality pathology in patients with Axis I disorders has been reported as a risk factor for poor treatment response and suicidal tendencies [53]. Predictive validity should be studied by examining the association between IPO scores and patient responses to drugs or psychotherapy.

It has often been noted that people with borderline psychopathology are more likely to have been victims of childhood adversities such as poor parenting and child abuse. This was proven to be the case in the present study. All the IPO subscale scores were associated with poor parenting styles and child abuse experiences.

These findings all suggest convergent and predictive validity of the IPO subscales. Because the factor structure study was cross-sectional in the present investigation, future studies should assess how stable the IPO-measured traits are in the long term. This may be linked to the possible influence of mood. Another important issue is potential cultural influence on the responses to the IPO. For example, an anthropologist, Nakane, said 'The vertical relation, which we predicted in theory from the ideals of social group formation in Japan, becomes the actuating principle in creating cohesion among group members' [54]. In such a society group membership is homogeneous and requires harmony as an important element of human life. Subordinates are expected to respect a superior. The superior is supposed to 'understand' what the subordinate wants to express without explicit communication. The unique structure of interpersonal competence was reported [55]. Such characteristics of the Japanese culture may determine the expression of borderline personality traits. International comparison of personality disorders and personality pathologies should be undertaken.

## Conclusion

The present studies have shown that the Japanese version and its subscales share a very similar factor structure with the original English version, and possess substantial validity.

This preliminary report suggests the potential utility of the Japanese version of the IPO in clinical and non-clinical populations. If future studies agree with our findings, the Japanese version of the IPO will prove an excellent tool to screen for personality pathology in busy psychiatric clinics, to measure responses to therapy, and, in an epidemiological setting, to identify subjects who require a structured interview for personality disorders.

## Competing interests

The authors declare that they have no competing interests.

## Authors' contributions

HI was a co-designer of the study and drafted the manuscript. HK, RK, and HM participated in data collection and processing. MS gave statistical advice. CH gave advice from psychoanalytical view points. TK was the main designer of the study and gave final approval to the published version.
